# Vaccination with a Recombinant H7 Hemagglutinin-Based Influenza Virus Vaccine Induces Broadly Reactive Antibodies in Humans

**DOI:** 10.1128/mSphere.00502-17

**Published:** 2017-12-13

**Authors:** Daniel Stadlbauer, Arvind Rajabhathor, Fatima Amanat, Daniel Kaplan, Abusaleh Masud, John J. Treanor, Ruvim Izikson, Manon M. Cox, Raffael Nachbagauer, Florian Krammer

**Affiliations:** a Department of Microbiology, Icahn School of Medicine at Mount Sinai, New York, New York, USA; b Department of Biotechnology, University of Natural Resources and Life Sciences, Vienna, Austria; c Center for Excellence in Youth Education, Icahn School of Medicine at Mount Sinai, New York, New York, USA; d Department of Medicine, University of Rochester Medical Center, Rochester, New York, USA; e Protein Sciences Corporation, Meriden, Connecticut, USA; University of Maryland School of Medicine

**Keywords:** H7N9, HA stalk, influenza, influenza virus vaccine

## Abstract

Human influenza virus infections with avian subtype H7N9 viruses are a major public health concern and have encouraged the development of effective H7 prepandemic vaccines. In this study, baseline and postvaccination serum samples of individuals aged 18 years and older who received a recombinant H7 hemagglutinin vaccine with and without an oil-in-water emulsion (SE) adjuvant were analyzed using a panel of serological assays. While only a small proportion of individuals seroconverted to H7N9 as measured by the conventional hemagglutination inhibition assay, our data show strong induction of anti-H7 hemagglutinin antibodies as measured by an enzyme-linked immunosorbent assay (ELISA). In addition, cross-reactive antibodies against phylogenetically distant group 2 hemagglutinins were induced, presumably targeting the conserved stalk domain of the hemagglutinin. Further analysis confirmed an induction of stalk-specific antibodies, suggesting that epitopes outside the classical antigenic sites are targeted by this vaccine in the context of preexisting immunity to related H3 hemagglutinin. Antibodies induced by H7 vaccination also showed functional activity in antibody-dependent cell-mediated cytotoxicity reporter assays and microneutralization assays. Additionally, our data show that sera from hemagglutination inhibition seroconverters conferred protection in a passive serum transfer experiment against lethal H7N9 virus challenge in mice. Interestingly, sera from hemagglutination inhibition nonseroconverters also conferred partial protection in the lethal animal challenge model. In conclusion, while recombinant H7 vaccination fails to induce measurable levels of hemagglutination-inhibiting antibodies in most subjects, this vaccination regime induces homosubtypic and heterosubtypic cross-reactive binding antibodies that are functional and partly protective in a murine passive transfer challenge model.

**IMPORTANCE** Zoonotic infections with high case fatality rates caused by avian H7N9 influenza viruses have been reported since early 2013 in China. Since then, the fifth wave of the H7N9 epidemic emerged in China, resulting in higher numbers of laboratory-confirmed cases than in previous years. Recently, H7N9 has started to antigenically drift and split into two new lineages, the Pearl River Delta and Yangtze River Delta clades, which do not match stockpiled H7 vaccines well. Humans are immunologically naive to these subtypes, and an H7N9 strain that acquires the capability of efficient human-to-human transmission poses a credible pandemic threat. Other characteristics of H7N9 are raising concerns as well, like its ability to bind to receptors in the human upper respiratory tract, the recent emergence of highly pathogenic variants, and the ability to quickly gain resistance to neuraminidase inhibitors. Therefore, developing and testing H7N9 vaccines constitutes a priority for pandemic preparedness.

## Abstract (precis)

Zoonotic infections with high case fatality rates caused by avian H7N9 influenza viruses have been reported since early 2013 in China. Since then, the fifth wave of the H7N9 epidemic emerged in China, resulting in higher numbers of laboratory-confirmed cases than in previous years. Recently, H7N9 has started to antigenically drift and split into two new lineages, the Pearl River Delta and Yangtze River Delta clades, which do not match stockpiled H7 vaccines well. Humans are immunologically naive to these subtypes, and an H7N9 strain that acquires the capability of efficient human-to-human transmission poses a credible pandemic threat. Other characteristics of H7N9 are raising concerns as well, like its ability to bind to receptors in the human upper respiratory tract, the recent emergence of highly pathogenic variants, and the ability to quickly gain resistance to neuraminidase inhibitors. Therefore, developing and testing H7N9 vaccines constitutes a priority for pandemic preparedness.

## INTRODUCTION

In addition to circulating human seasonal influenza virus strains, avian influenza A (H7N9) viruses emerged as a public health concern in 2013 (1). H7N9 viruses frequently cause severe lower respiratory tract infections in humans in China but have not yet gained the capability of sustained human-to-human transmission (2). During the recent 2016–2017 Northern Hemisphere winter season, the fifth wave of the H7N9 epidemic hit China, causing more cases than in previous years. Currently, over 1,500 laboratory-confirmed cases of H7N9 with a case fatality rate of almost 40% have been reported (3). During the fifth wave, a highly pathogenic variant (for poultry) of the H7N9 virus which features a polybasic cleavage site in hemagglutinin (HA) emerged (4, 5). In addition, H7N9 has split into two antigenic lineages, the Pearl River Delta (PRD) and the Yangtze River Delta (YRD) lineages, which have been shown to not match H7N9 stockpiled vaccines well when tested with ferret antisera (4). If the avian virus either adapts to humans through mutations or undergoes reassortment with seasonal influenza virus strains circulating in the human population (6, 7), H7N9 could gain pandemic potential (8). Therefore, it is important to have a good understanding of the human immune response to the H7 HA and H7 vaccines that are being developed for pandemic preparedness.

Humoral responses to influenza virus vaccine candidates are traditionally evaluated in a hemagglutination inhibition (HI) assay. Humans are immunologically naive to the H7N9 subtype and have very low baseline immunity and HI titers (9). The HI assay measures titers of strain-specific antibodies binding to the HA head domain which inhibit binding of the HA to sialylated host receptors by steric hindrance (10, 11). In human trials, a serum HI antibody titer of ≥1:40 was established as a correlate of protection from seasonal influenza viruses and is now used as a criterion for vaccine licensure (11, 12). However, it is unclear if this surrogate of protection is adequate for avian influenza virus strains. Additionally, antibodies that bind the highly conserved HA stalk are not detected in this assay, because they do not interfere with receptor binding. These HA stalk antibodies were previously shown to be broadly cross-reactive against multiple influenza virus strains. While cross-group HA stalk binding antibodies exist, most stalk-reactive antibodies are restricted in binding to either group 1 HAs (H1, H2, H5, H6, H8, H9, H11, H12, H13, H16, HA-like H17, and HA-like H18) or group 2 HAs (H3, H4, H7, H10, H14, and H15) (13–15). Cross-reactive stalk-based antibodies neutralize the virus by binding to the membrane-proximal stalk domain and prevent infection by inhibiting the fusion of viral and endosomal membranes or reducing viral titers by other mechanisms like Fc-mediated effector functions (antibody-dependent cell-mediated cytotoxicity [ADCC], antibody-dependent cellular phagocytosis [ADCP], or complement-dependent lysis [CDL]). These antibodies also show neutralizing activity *in vitro* (although at a lower potency than HI-active antibodies) and confer protection *in vivo* (11, 16, 17). In the present study, we analyzed the titers, breadth, functionality, and protective efficacy of antibodies induced by two doses of a prepandemic recombinant H7 HA vaccine in humans. Information about the potential to elicit broad antibody responses could aid the development of novel universal or broadly protective influenza virus vaccine candidates and guide pandemic preparedness efforts directed against emerging influenza viruses (18–20).

## RESULTS

### Recombinant H7 vaccination induces robust anti-H7 binding antibody titers.

**FIG 1:**
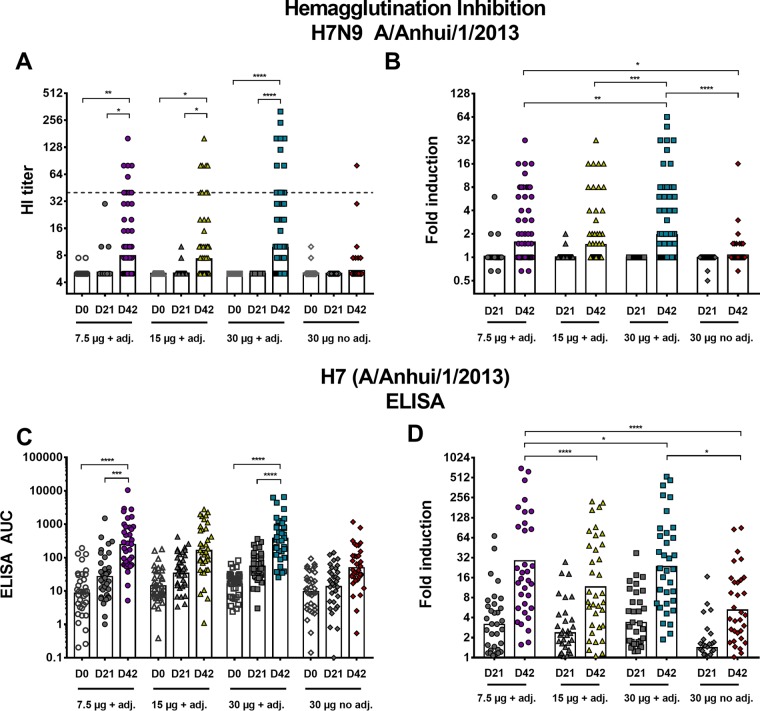
Human antibody response to vaccination with recombinant H7 HA as measured by HI assay (A and B) and ELISA (C and D). (A) HI titers of enrolled subjects (*n* = 382) at time points day 0 (D0), day 21, and day 42 postprime for the four different treatment groups. The dashed line represents an HI titer of 1:40, which was defined as seroconversion (4-fold increase in HI titer or HI titer of ≥1:40). The bars indicate the geometric mean (GM) of all data points. (B) Induction of HI titers over baseline after one vaccination (D21) and two vaccinations (D42). (C) Absolute ELISA AUC values of antibodies binding to matched HA of A/Anhui/1/2013 after vaccination with recombinant H7 HA. (D) Induction for the time points day 21 and day 42 postvaccination for the four different treatment groups. The results are presented as GM values relative to baseline. In panels A and C, time points day 0, day 21, and day 42 were compared to each other within a treatment group in a one-way ANOVA. In panels B and D, each day 21 time point was compared to every day 21 time point of all other treatment groups. The same comparison was performed for the day 42 time point. Significance is indicated as follows: no symbol, *P* > 0.05; *, *P* ≤ 0.05; **, *P* ≤ 0.01; ***, *P ≤* 0.001; ****, *P ≤* 0.0001. adj., adjuvant.

Healthy subjects received two doses of a recombinant monovalent full-length H7 HA vaccine intramuscularly 21 days apart. In this study, 407 subjects were enrolled. Out of those, 382 met the evaluable criteria, which were defined as two immunizations and serology draws at two predefined time points (days 0 and 42 postprime). Additionally, blood was drawn at day 21 postvaccination. The 407 participants were split up into four different treatment groups. One group received 30 µg of nonadjuvanted recombinant HA, and the other three groups received various amounts of recombinant HA (7.5, 15, or 30 µg) adjuvanted with a 2% stable oil-in-water emulsion (SE) (21). Only 36 (9.4%) of the 382 evaluable individuals seroconverted (≥1:40) to H7N9 as measured by the conventional hemagglutination inhibition (HI) assay (Fig. 1A and B). An even distribution of seroconverters, subjects with a rise from baseline not meeting the seroconversion definition, and subjects with no change from baseline were randomly selected for further analysis (*n* = 35 per treatment group) (see Fig. S1A in the supplemental material). Our data show strong induction of anti-H7 HA antibodies by enzyme-linked immunosorbent assay (ELISA) (Fig. 1C and D). Only low induction of antibodies was observed after one vaccination for all groups (3.2-fold [95% confidence interval {CI}, 2.2 to 4.6] for 7.5 µg plus adjuvant, 2.4-fold [95% CI, 1.7 to 3.3] for 15 µg plus adjuvant, 3.7-fold [95% CI, 2.4 to 4.7] for 30 µg plus adjuvant, and 1.4-fold [95% CI, 1.1 to 1.8] for 30 µg, nonadjuvanted). For the 7.5-µg recombinant HA adjuvanted group, an induction of 28.6-fold (95% CI, 14.7 to 55.5) over baseline was measured after 2 vaccinations at day 42. For the 15-µg recombinant HA adjuvanted group, an induction of 11.5-fold was detected (95% CI, 6.5 to 20.4), and for the 30-µg recombinant HA adjuvanted group, an induction of 23.3-fold was detected (95% CI, 13.1 to 41.4). The nonadjuvanted group (30-µg recombinant HA) showed much lower induction of 5.2-fold (95% CI, 3.3 to 8.1) at day 42 postprime. This highlights the need for the administration of at least two doses of the vaccine and shows that the addition of adjuvant increases the immunogenicity, leading to higher titers of measurable binding antibodies. No clear dose dependence was observed. In fact, the induction was highest (28.6-fold) for the lowest-dose (7.5 µg plus adjuvant) recombinant HA group within the subselection of samples (*n* = 35).

10.1128/mSphere.00502-17.1 FIG S1  Flow chart of sample subselection. The number of subjects (*n*) tested in the different assays is depicted. Download FIG S1, TIF file, 0.5 MB. Copyright © 2017 Stadlbauer et al. 2017 Stadlbauer et al. This content is distributed under the terms of the Creative Commons Attribution 4.0 International license.

### Antibodies induced by vaccination with recombinant H7 HA from the A/Anhui/1/2013 H7N9 strain bind to HAs of emerging H7 viruses.

**FIG 2:**
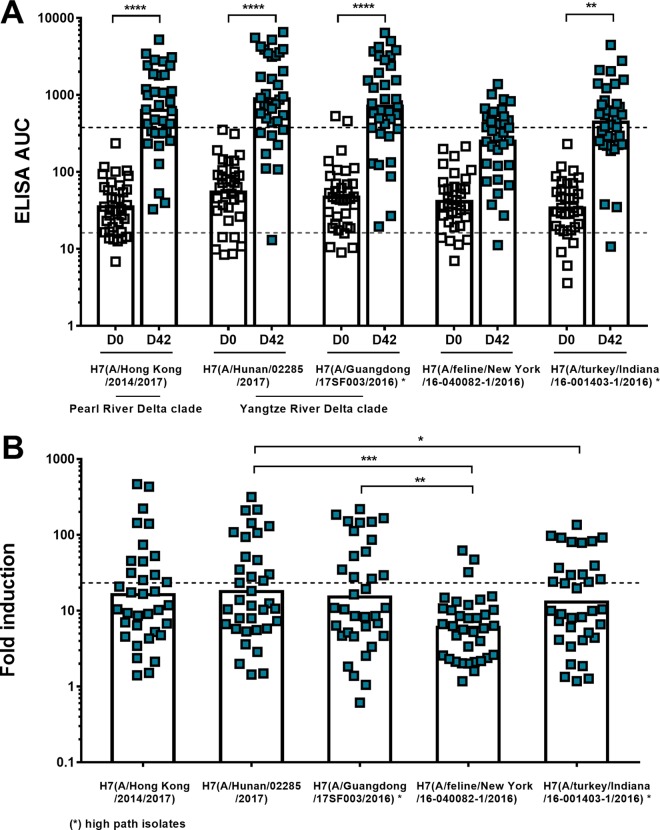
Cross-reactive antibody response to HAs from emerging Eurasian and American lineage H7 viruses after H7 A/Anhui/1/2013 vaccination as measured by ELISA. (A) Serum samples from a subselection of samples (high-dose 30-µg recombinant HA adjuvanted group) were tested for binding to H7 HAs of different H7NX virus isolates (H7N9 A/Hunan/02285/2017, H7N9 A/Hong Kong/2014/2017, H7N9 A/Guangdong/17SF003/2016, H7N2 A/feline/New York/16-040082-1/2016, and H7N8 A/turkey/Indiana/16-001403-1/2016). Absolute ELISA area under the curve (AUC) values were determined. Data for baseline (D0, white) and postvaccination (D42, red) serum samples are shown. The dashed lines represent the GM titer of serum antibodies binding to A/Anhui/1/2013 at day 0 (gray line) and day 42 (black line) as shown in Fig. 1C. (B) Fold induction of cross-reactive H7 antibodies based on ELISA AUC values postvaccination (D42). The dashed line represents the induction based on the ELISA AUC values of serum antibodies binding to A/Anhui/1/2013 as shown in Fig. 1D. Time points day 0 and day 42 were compared within each treatment group in a one-way ANOVA. Significance is indicated as follows: no symbol, *P* > 0.05; *, *P* ≤ 0.05; **, *P* ≤ 0.01; ***, *P ≤* 0.001; ****, *P ≤* 0.0001.

Cross-reactivity of antibodies induced by recombinant HA vaccination within subtype H7 HAs was determined by performing ELISAs with HAs from viruses that emerged in 2016 and 2017 from both the Eurasian and North American H7 lineages. Testing was restricted to sera from a subselection of subjects (*n* = 35) of the high-dose (30-µg) adjuvanted treatment group. It is of interest to know if the antibodies induced by the vaccine strain of 2013 are reactive to drifted, evolving strains from both the Pearl River Delta (PRD) and Yangtze River Delta (YRD) lineages that are currently found in infected humans in China. Additionally, it was investigated if there is cross-reactivity to an H7 HA from the North American lineage highly pathogenic avian H7N8 virus as well as to the H7 of an H7N2 feline virus strain that led to an outbreak in cats (with one human zoonotic event) in an animal shelter in New York City (22–24). Our data showed that there was a 16.2-fold induction of binding to A/Hong Kong/2014/2017 (Hong Kong, PRD) HA, a 17.9-fold induction of binding to A/Hunan/02285/2017 (Hunan, YRD) HA, and a 15.2-fold induction of binding to A/Guangdong/17SF003/2016 (Guangdong, YRD, highly pathogenic isolate) HA after vaccination (Fig. 2A and B). The increase of antibodies that bound to the North American lineage H7N2 feline virus HA (6.0-fold increase at day 42 for A/feline/New York/16-040082-1/2016) and to H7 from the avian H7N8 A/turkey/Indiana/16-001403-1/2016 virus isolate (13.0-fold induction at day 42) was lower, likely due to their larger phylogenetic distance to the A/Anhui/1/2013 strain (Eurasian lineage). These data indicate persistent reactivity of induced antibodies to emerging H7 viruses of both lineages.

### Recombinant H7 HA vaccination induces antibodies that cross-react to all other group 2 HAs.

**FIG 3:**
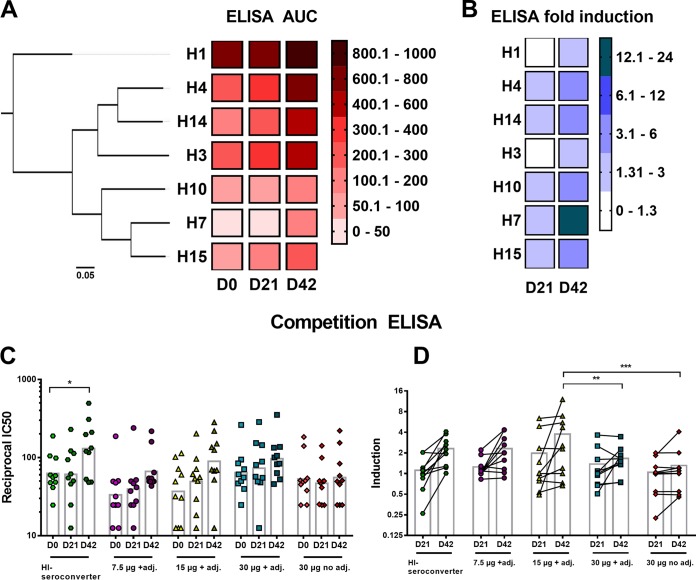
Cross-group reactivity. (A) A phylogenetic tree of group 2 HAs is depicted on the left. The scale bar at the bottom shows 5% difference in amino acid identity. Heat map showing the ELISA area under the curve values for H1, H4, H14, H3, H10, H7, and H15 for time points day 0 (D0), day 21 (D21), and day 42 (D42) postvaccination. The color key (AUC values of 0 to 1,000) is shown on the right. For analysis, all treatment groups (*n* = 35 per group) were combined. (B) Heat map representing the fold induction based on absolute ELISA values over baseline at time points day 21 (D21) and day 42 (D42) postvaccination for all group 2 HAs and group 1 HA H1 (Cal09). The color key is shown (fold induction of 0 to 24) on the right. The four different treatment groups were combined for analysis (*n* = 35 per group). (C) Reciprocal IC_50_ values for the five different groups as measured in an H3/stalk mAb competition ELISA are shown for time points day 0, day 21, and day 42. The bars represent the geometric mean values. Time points day 0, day 21, and day 42 were compared to each other within a treatment group in a one-way ANOVA. (D) Induction of reciprocal IC_50_ values for day 21 and day 42 serum samples over baseline (day 0). The bars show the GM values. Statistical significance was analyzed for each day 21 time point compared to every other day 21 time point. The same procedure was applied for time point day 42. Significance is indicated as follows: no symbol, *P* > 0.05; *, *P* ≤ 0.05; **, *P* ≤ 0.01; ***, *P ≤* 0.001; ****, *P ≤* 0.0001. adj., adjuvant.

The antibody response to HAs from all other group 2 subtypes (H3, H4, H10, H14, and H15) and to H1 HA (A/California/4/2009 [Cal09]) was measured at three time points (days 0, 21, and 42), and geometric mean (GM) titers of all treatment groups combined are shown as a heat map (Fig. 3A). The area under the curve (AUC) values, measured by ELISA, are high for the H3 clade HAs (H3, H4, and H14) at day 0, which is likely caused by preexisting antibodies to the globally circulating seasonal H3N2 influenza A virus strains (9, 25). The participants in the vaccine trial were 18 years and older and had most likely previously been exposed to multiple H3N2 viruses (26). The AUC values for the H7 clade HAs (H7, H10, and H15) were low on day 0, and they were boosted only to levels that reach the H3 clade AUC values at baseline. The low antibody levels that were detected on day 0 for the H7 clade HAs were presumably antibodies that bind to the highly conserved HA stalk domain. Low baseline titers of H7 clade HA (H7, H10, and H15) antibodies in humans, including the absence of head-specific immunity to H7 virus strains, have previously been reported (9, 27, 28). For the group 1 hemagglutinin H1 (Cal09), the AUC values were higher on day 0 than H3 HA baseline AUC values, which suggests a strong preexposure to H1N1 and/or other group 1 HA viruses (9). The fold induction over baseline on day 42, based on the ELISA AUC values, was highest (14.2-fold) for H7 HA compared to the other HAs (Fig. 3B). The antibodies reactive to the two other H7 clade HAs—H10 and H15—increased by 3.5-fold and 5.0-fold, respectively. The induction of antibodies binding to the H3 clade HAs was lower (3.1-fold for H4, 2.9-fold for H14, and 1.9-fold for H3), most likely because they are phylogenetically more distant from H7 and because baseline antibody levels were higher. For H1, a fold induction of 1.3 over baseline on day 42 was detected, possibly mediated by the induction of cross-group-reactive stalk-specific antibodies (25, 29, 30).

To confirm that the observed cross-reactive responses were actually mediated by an increase of group 2 stalk-specific antibodies, competition ELISAs for H3 with the well-characterized stalk binding mouse monoclonal antibody (MAb) 9H10 (31) were performed (Fig. 3C and D). Sera from 10 individuals per treatment group and sera from 10 subjects who seroconverted by HI (see below) were randomly selected. Preincubation of H3 with human serum resulted in a decrease of binding of MAb 9H10 (see Fig. S2 in the supplemental material) to H3. 9H10-competing serum antibody levels were indirectly measured and are depicted as reciprocal half-maximal inhibitory concentration (IC_50_). The increase of geometric mean (GM) IC_50_s over baseline (Fig. 3D) after one vaccination at day 21 was 0.98-fold for the HI seroconverter group, 1.19-fold for 7.5 µg plus adjuvant, 1.34-fold for 15 µg plus adjuvant, 1.14-fold for 30 µg plus adjuvant, and 0.88-fold for the 30-µg unadjuvanted group. After two vaccinations (day 42), a higher increase of GM IC_50_s could be observed (2.09-fold for the HI seroconverters, 2.00-fold for the 7.5-µg-plus-adjuvant group, 2.43-fold for the 15-µg-plus-adjuvant group, 1.51-fold for the 30-µg-plus-adjuvant group, and 1.05-fold for the 30-µg unadjuvanted group). The competing serum antibodies measured in this assay are not representative of the whole repertoire of stalk-specific serum antibodies. Only antibodies with the same epitope as MAb 9H10, or epitopes close to the footprint of 9H10, are being detected.

10.1128/mSphere.00502-17.2 FIG S2  Competition ELISA (A to E) shows percent inhibition for day 0, day 21, and day 42 sera at serial dilutions for the five different groups analyzed in the experiment. The dotted lines represent 0% and 100% inhibition. Download FIG S2, TIF file, 0.5 MB. Copyright © 2017 Stadlbauer et al. 2017 Stadlbauer et al. This content is distributed under the terms of the Creative Commons Attribution 4.0 International license.

### Antibodies induced by H7 vaccination are functional *in vitro* and protective *in vivo*.

**FIG 4:**
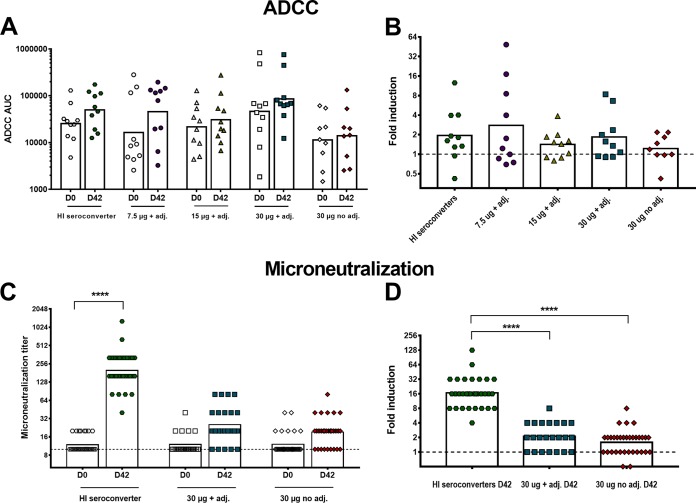
*In vitro* functionality of human serum antibodies induced by H7 vaccination. (A) ADCC AUC values measured for the four different treatment groups and the HI seroconverter group. The bars show the geometric means of the AUC values. (B) GMs of induction over baseline at day 42 after two vaccinations based on AUC values are represented for the HI seroconverter group (1.9-fold, green hexagons), 7.5-µg-plus-adjuvant group (2.8-fold, purple circles), 15-µg-plus-adjuvant (adj.) group (1.4-fold, yellow triangles), 30-µg-plus-adjuvant group (1.8-fold, blue squares), and 30-µg no-adjuvant group (1.2-fold, red diamonds). The dashed line represents a 1-fold increase in ADCC activity. (C) Individual titers of neutralizing serum antibodies in a microneutralization assay using the H7N9 A/Shanghai/1/2013 strain. The HI seroconverter group (green hexagons), high-dose (30-µg) adjuvanted group (blue squares), and 30-µg nonadjuvanted group (red diamonds) were selected for analysis. The dashed line represents the limit of detection (titer of 1:10), and the bars show the geometric means. (D) Increase in microneutralization (MN) titers over baseline at day 42. The dashed line represents a 1-fold increase in microneutralization titers. In panels A and C, time points day 0 and day 42 within one group were analyzed for significance values in a one-way ANOVA. In panels B and D, each column was compared to every other column. Significance is indicated as follows: no symbol, *P* > 0.05; *, *P* ≤ 0.05; **, *P* ≤ 0.01; ***, *P ≤* 0.001; ****, *P ≤* 0.0001.

To test the functional activity of antibodies induced by H7 vaccination, ADCC reporter and microneutralization assays were performed. Protectivity *in vivo* was assessed in a murine passive transfer challenge model. For the ADCC reporter assay, microneutralization, and passive serum transfer challenge experiments, HI seroconverters (34 subjects) were removed from all four initial groups and defined as an individual fifth group (Fig. S1). The seroconverters were excluded to be able to tease out differences in antibody responses to H7 HA between seroconverters and nonconverters in different assays. Sera from 10 individuals per group, as mentioned above, were tested in an ADCC reporter assay. We observed a slight increase in ADCC reporter activity after recombinant H7 vaccination for all groups (Fig. 4A). Again, the 7.5-µg-plus-adjuvant group had the highest induction of activity (2.8-fold [95% CI, 1.0 to 8.0]), as measured in the ADCC bioreporter assay (Fig. 4B), followed by the newly defined HI seroconverter group (1.9-fold [95% CI, 1.0 to 3.8]). The increase in activity for the other groups, 15 µg plus adjuvant, 30 µg plus adjuvant, and 30 µg unadjuvanted, was lower with fold inductions of 1.4 (95% CI, 1.0 to 2.0), 1.8 (95% CI, 1.0 to 3.3), and 1.2 (95% CI, 0.8 to 1.8), respectively. It must be noted that the spread of values was high in all groups. Therefore, it is not possible to draw definite conclusions.

Subsequently, microneutralization assays were performed with pre- (day 0) and postvaccination (day 42) sera of the HI seroconverter, 30-µg-plus-adjuvant, and 30-µg unadjuvanted groups using the A/Shanghai/1/2013 strain, which is closely related to the vaccine strain and was used for technical reasons (Fig. 4C). The sera from the HI seroconverter group had the highest increase (16.7-fold) in microneutralization titers, whereas the titers for the high-dose adjuvanted group increased 2.1-fold and those for the unadjuvanted group without seroconverters increased 1.6-fold (Fig. 4D). These results indicate that neutralization is mostly, but not exclusively, mediated by HI-active antibodies that bind to the receptor binding site and thereby prevent viral attachment to host cell receptors (10, 11).

**FIG 5:**
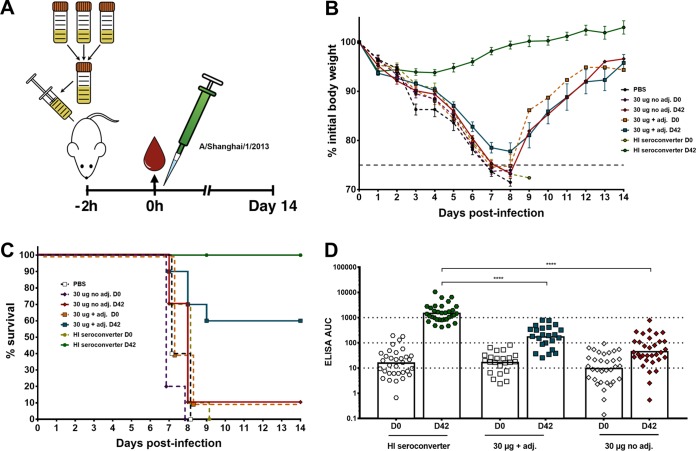
*In vivo* protectivity of human serum antibodies in a lethal mouse challenge model. (A) Graphic representation of the serum transfer and lethal challenge animal experiment. Sera from all individuals of a selected group were pooled and given to female BALB/c mice (*n* = 10 per group). After 2 h, the mice were infected intranasally with 5 50% lethal doses (2 × 10^4^ PFU) of H7N9 virus (A/Shanghai/1/13, PR8-based 6:2 reassortant). Weight loss and survival were monitored for 14 days. (B) The weight loss curve of the mouse challenge experiment is shown. The dashed colored lines represent the prevaccination sera. The dashed gray line represents 75% of initial body weight, which was used as the humane endpoint. (C) Survival graph showing percent survival in the different groups used in the animal experiment. The dashed lines represent prevaccination sera. (D) Anti-H7 antibody levels of the groups used in the challenge experiment are shown as ELISA AUC values. The dotted lines at titers of 10, 100, and 1,000 indicate differences of 1 log. Time point day 0 of each group was tested against every other time point day 0 in a one-way ANOVA with a Sidak posttest for multiple comparisons. The same analysis was applied for time point day 42. Significance is indicated as follows: no symbol, *P* > 0.05; *, *P* ≤ 0.05; **, *P* ≤ 0.01; ***, *P ≤* 0.001; ****, *P ≤* 0.0001. adj., adjuvant.

Next, the protective efficacy of the vaccine-induced antibodies was determined *in vivo* in a murine passive transfer challenge model. Day 0 and day 42 serum pools were generated for each of the selected subsets of samples and transferred into mice via intraperitoneal injection. After 2 h, the mice were infected intranasally with an H7N9 (A/Shanghai/1/2013) challenge virus (Fig. 5A). This virus is an A/Puerto Rico/8/34 (PR8)-based reassortant virus that consists of the six internal segments of PR8 and the HA and NA of the A/Shanghai/1/2013 isolate. The HA of this virus shares 98% amino acid sequence identity with the HA of A/Anhui/1/2013 and was selected because it induces morbidity and mortality in the mouse model (32). Sera from the HI seroconverter postvaccination group (day 42) conferred full protection from lethal H7N9 challenge (Fig. 5B), whereas mice that received the postvaccination sera from the high-dose adjuvanted group (without seroconverters) were partially protected. The mice showed morbidity, but 6 out of 10 mice recovered (Fig. 5C). All mice that received prevaccination sera (day 0) and mice that received the high-dose nonadjuvanted postvaccination (day 42) sera succumbed to infection at day 7 or 8 postinfection, except for one survivor in the 30-µg nonadjuvant postvaccination group and one in the 30-µg adjuvanted prevaccination group (Fig. 5C). These data indicate that the sera from the HI seroconverters, which also had the highest overall anti-H7 antibody levels (Fig. 5D) measured by ELISA, contain functionally active antibodies that are protective in an animal challenge model. However, sera from HI nonseroconverters also conferred partial protection, likely mediated by mechanisms based on antibody effector functions like ADCC, ADCP, and/or complement-dependent cytotoxicity in addition to HI-active antibodies below the limit of detection in the HI assay. Additionally, neutralization might be mediated by antibodies binding to the membrane-proximal HA stalk domain (10, 11, 17). The ELISA data corresponding to the groups in the challenge experiment showed that postvaccination geometric mean ELISA AUC values (against the matched H7 HA antigen) of approximately 1,000 (HI seroconverters) conferred full protection, whereas values of approximately 100 (high-dose adjuvant group) conferred partial protection and AUC values below 100 (high-dose nonadjuvanted) conferred no protection *in vivo* (Fig. 5D).

## DISCUSSION

Avian H7N9 viruses are a risk to public health and pose a potential pandemic threat. Besides the large number of human cases, several attributes of H7N9 influenza viruses are raising concerns, including their ability to bind to human receptor orthologs (33), to quickly gain resistance to neuraminidase inhibitors under treatment without substantial loss of fitness (34), and to often asymptomatically spread among poultry (22). They also have acquired mutations that are facilitating growth in human tissues (35), and there is evidence of limited human-to-human transmission within family clusters through direct contact (36) and for transmission via aerosol exposure in a ferret model (37). Therefore, it is important to develop and test H7 vaccines that can be used as a preventive measure to rapidly respond to a potential pandemic. It has been previously shown that H7 vaccines often fail to induce seroconversion (HI titer of ≥1:40 or 4-fold increase in HI titers postvaccination) (28, 38, 39). However, it remains unclear what level of HI titer is associated with protection against emerging influenza virus strains, including the H7N9 subtype. A serum HI antibody titer of ≥1:40 was defined as a correlate of protection from seasonal influenza viruses, but whether this also applies to avian zoonotic virus strains is unclear (10). Additionally, the H7 HA seems to have a less immunodominant head domain, leading to the preferential induction of antibodies directed against nonclassical antigenic sites of the HA and consequently a lower HI titer (10, 28, 29, 38). Humans have negligible preexisting head-specific HI-based immunity to nonseasonal influenza virus strains. Upon exposure to such virus strains, responses to the conserved HA stalk domain, which hosts epitopes shared with seasonal influenza virus strains, are preferably elicited (18, 19, 40, 41).

Recently, it was shown that H7N9 vaccination induces both group-specific and cross-group-reactive HA stalk binding B cells (29). In the present study, we detected a high induction of anti-H7 antibodies in an ELISA in individuals without HI seroconversion. A portion of these antibodies were broadly cross-reactive, and we detected binding to all other group 2 HAs (H3, H4, H14, H10, and H15). These cross-reactive responses were likely mediated by stalk-specific cross-reactive group 2 antibodies (26, 42, 43) as confirmed in a stalk-based competition ELISA with titers that increased noticeably after vaccination. The induction of antistalk antibodies was more apparent in the adjuvanted groups. It is unlikely that the use of adjuvant redirected the antibody response to the stalk domain. However, the stronger immune response induced by the adjuvanted vaccine might have made the presence of these antibodies more apparent and easier to detect. Interestingly, insect cell-produced recombinant HA vaccines have in the past been shown to induce broader protection than classical, egg-derived vaccines (44). It has been hypothesized that this might be caused by the smaller glycan size of insect cell-derived HA (45), which might allow a higher accessibility of stalk epitopes that are shielded by conserved glycans (46, 47). However, due to the lack of an egg-derived comparator vaccine, this hypothesis could not be tested in the current study. The vaccinees also exhibited heterologous cross-reactivity within the H7 HA subtype. Tested individuals showed high antibody levels measured by ELISA to HAs of three novel H7N9 virus isolates from China (Eurasian lineage), of one North American H7N2 cat virus isolate, and of one North American avian high-pathogenicity H7N8 isolate. This demonstrates the potential of a vaccine against the A/Anhui/1/2013 strain to elicit cross-reactive antibodies against novel, evolving H7N9 viruses and zoonotic H7NX viruses.

Furthermore, the *in vitro* functionality and *in vivo* protective efficacy of these antibodies were demonstrated. Sera from the HI seroconverter group showed the highest levels of antibodies measured by ELISA and microneutralization and conferred full protection in the animal H7N9 challenge model. However, we also found that sera from HI nonseroconverters conferred partial protection from mortality in a serum transfer mouse model. In this subset, protection might have been mediated by stalk-reactive antibodies and Fc-mediated effector functions like ADCC, ADCP, and CDL even though only low levels of ADCC activity were detected in a reporter assay (17, 48). The protective efficacy mediated by human nonneutralizing antibodies might be lower in mice than in humans even though the binding affinities of human IgG to murine Fc receptors and human IgG to human Fc receptors are somewhat similar (49). As demonstrated, a big part of the immune response to vaccination can be overlooked when only HI titers are taken into account. This further emphasizes the need to develop additional correlates of protection for influenza virus infections. Natural infection with H7N9 viruses has been shown to elicit strong humoral responses, including HI titers in humans (25, 30), but so far it has not been possible to mimic these responses by vaccination schemes with either live attenuated influenza vaccines (LAIV), inactivated influenza vaccines (IIV), or recombinant H7 HA formulations (27, 28, 38, 39, 50–53). Other vaccine candidates, e.g., those based on virus-like particles (VLPs) (54), could not elicit robust immune responses to H7 either, as shown in past clinical trials in humans.

Additionally, it has become clear that, in the absence of an H7-primed immune system, at least two vaccinations with any H7N9 vaccine are necessary (53) and that vaccine formulations need to be supplemented with strong adjuvants to boost immune responses to high levels (55). This is reminiscent of H5 vaccines, which also show lower immunogenicity in humans than seasonal influenza virus vaccines (56, 57). However, the immunogenicity of H5-based vaccines seems to be higher than that of H7-based vaccines. Further development of new vaccination strategies, like DNA or LAIV prime followed by boosting with monovalent inactivated virus vaccines (52, 53, 58, 59) or strategies based on mRNA administration (60), and enhanced understanding of the immune response to emerging viruses are needed to tackle pandemic threats. Moreover, the addition of recombinant influenza virus neuraminidase to recombinant HA-based but also conventional inactivated vaccines could add another independent path for protection against influenza virus infections (61).

## MATERIALS AND METHODS

### Vaccine.

The vaccine consists of monovalent pandemic H7N9 recombinant HA influenza virus vaccine derived from A/Anhui/1/2013 manufactured in the baculovirus expression vector system (62). The unadjuvanted formulation consists of recombinant HA alone, while the adjuvanted formulations were mixed at a 1:1 ratio with a stable oil-in-water emulsion (SE). Recombinant HA content for formulation was determined by the single radial immunodiffusion assay. The antigen was stored in sodium phosphate buffer with 0.005% Tween 20, pH 7.0, and 0.01% thimerosal. A final 0.36-ml dose of adjuvanted recombinant HA supplemented with SE or unadjuvanted recombinant HA was administered intramuscularly.

SE (Infectious Disease Research Institute, Seattle, WA) is an oil-in-water formulation that appears as a milky-white emulsion. The emulsion contains squalene (oil), glycerol, phosphatidylcholine, surfactant (poloxamer), and buffer (ammonium phosphate). Squalene is sourced from sharks; the other components are synthesized chemically.

### Cells, viruses, and proteins.

BTI-TN5B1-4 (*Trichoplusia ni*) cells were maintained in serum-free SFX medium (HyClone) supplemented with antibiotics (100 U/ml penicillin, 100 µg/ml streptomycin; Gibco). Madin-Darby canine kidney (MDCK) cells were grown in Dulbecco’s modified Eagle’s medium (DMEM; Gibco). DMEM was supplemented with a penicillin-streptomycin antibiotic mix (100 U/ml penicillin, 100 µg/ml streptomycin; Gibco) and fetal bovine serum (FBS, 10%; HyClone). Single-use aliquots of ADCC bioeffector FcγRIIIa cells (Promega) were thawed before usage. The H7N9 viruses were grown in 8- to 10-day-old embryonated chicken eggs (Charles River Laboratories) at 37°C for 48 h. The viral titers were determined by plaque assay using MDCK cells as previously described (41). The viruses consist of the HA and NA segments of the original virus isolates, A/Shanghai/1/2013 and A/Anhui/1/2013, respectively, combined with the backbone of the A/Puerto Rico/8/1934 (PR8) virus isolate. The recombinant proteins, including H1 from A/California/04/2009 virus; H3 from A/Hong Kong/4801/2014 virus; H4 from A/duck/Czech/1956 virus; H7 from A/Anhui/1/2013, A/Hunan/02285/2017, A/Guangdong/17SF003/2016, A/Hong Kong/2014/2017, A/feline/New York/16-040082-1/2016, and A/turkey/Indiana/16-001403-1/2016 virus; H10 from A/Jiangxi-Donghu/346/2013 virus; H14 from A/mallard/Gurjev/263/1982 virus; and H15 from A/shearwater/West Australia/2576/1979 virus, were produced in the baculovirus expression system as described before (63, 64). For the highly pathogenic avian A/Guangdong/17SF003/2016 and A/turkey/Indiana/16-001403-1/2016 virus isolates, the polybasic cleavage site was removed to increase recombinant protein stability, resulting in sequences with regular low-pathogenic avian influenza (LPAI) H7N9 cleavage sites.

### Human serum samples.

The tested human serum samples were obtained during a phase I/II double-blind, adaptive-design clinical trial to evaluate the immunogenicity and safety of Panblok, conducted with Protein Sciences Corporation’s recombinant pandemic H7 HA vaccine (ClinicalTrials.gov identifier NCT02464163). Informed consent was obtained from all 407 enrolled subjects, who were then randomized equally into four different treatment groups, receiving 7.5, 15, or 30 µg recombinant HA adjuvanted with 2.0% SE or 30 µg unadjuvanted recombinant HA twice intramuscularly. The participants were healthy adults aged 18 years or older. Serum samples (*n* = 35 per group) before vaccination (day 0) and after one (day 21) and two (day 42) vaccinations were provided as deidentified samples for analysis. The subselected samples contained an even distribution of seroconverters, subjects with a rise from baseline not meeting the seroconversion definition, and subjects with no change from baseline. Because the study was limited in the number of seroconverters and subjects with a rise from baseline, the samples chosen included almost all seroconverters and subjects with a rise from baseline. Subjects with no rise from baseline were chosen randomly. For the microneutralization assay, ADCC assay, and passive serum transfer challenge experiments, HI seroconverters were excluded from all four groups and defined as an individual fifth group (baseline and day 42). Seroconverters are defined as subjects with either a prevaccination HI titer of <1:10 and a postvaccination HI titer of >1:40 or a prevaccination titer of >1:10 and a minimum 4-fold rise in postvaccination HI antibody titer (as defined by the FDA). Subjects with HI titers below the limit of detection at baseline need HI titers of at least 1:40 postvaccination to be considered seroconverters.

### Hemagglutination inhibition assay.

HI antibody testing was carried out by a central laboratory (Southern Research Institute [SRI]) using a qualified assay that employed a whole-virus antigen. The influenza virus A/Anhui/1/2013 isolate was obtained from the Centers for Disease Control and Prevention (CDC) and amplified in eggs at SRI under appropriate biocontainment conditions. Serum samples were treated initially with receptor-destroying enzyme (RDE; Denka Seiken, Tokyo, Japan) to remove nonspecific inhibitors of hemagglutination. Sera were tested at an initial dilution of 1:10 (lower limit of detection [LOD] of the assay), with subsequent 2-fold serial dilutions (1:20, 1:40, 1:80, 1:160, etc.). The assays were performed using 1.0% equine red blood cells (RBCs; Lampire Biologicals) diluted in phosphate-buffered saline (PBS). Titers of 1:5 were assigned to HI-negative subjects to facilitate data analysis and data representation.

### ELISA.

Microtiter plates (96-well plates; Thermo Fisher) were coated with 50 µl of recombinant HA diluted to a concentration of 2 µg/ml in coating buffer (SeraCare) overnight at 4°C. The next day, the plates were blocked with 220 µl of blocking solution consisting of phosphate-buffered saline (PBS; pH 7.4; Gibco) supplemented with 0.1% Tween 20 (PBS-T), 3% goat serum (Life Technologies), and 0.5% milk powder (American Bio) for at least 1 h at room temperature. Human serum samples were diluted to a starting concentration of 1:100, serially diluted 1:2 in blocking solution, and incubated at room temperature for 2 h. The plates were washed three times with PBS-T, and 50 µl of secondary antibody, anti-human IgG (Fab specific) that was conjugated with horseradish peroxidase (HRP), produced in goat (Sigma catalog no. A0293), and diluted 1:3,000 in blocking solution, was added to each well. After 1 h, plates were washed four times with PBS-T. The plates were developed with SigmaFast *o*-phenylenediamine dihydrochloride (OPD; Sigma) for 10 min, and the reaction was stopped with 3 M HCl (Thermo Fisher). The plates were read at 490 nm with a microplate reader (BioTek). The data were analyzed in Microsoft Excel and GraphPad Prism 7, and the area under the curve (AUC) values were determined. The cutoff value was defined as the average of the values of blank wells plus 3 times the standard deviation of the blank wells.

### Competition ELISA.

Microtiter plates (96-well plates; Thermo Fisher) were coated with 50 µl of recombinant H3 A/Hong Kong/4801/2014 protein at a concentration of 2 µg/ml in coating buffer (KPL) overnight at 4°C. The following day, the plates were washed three times and blocked with 220 µl PBS-T per well for 1 h at room temperature. Human serum samples were diluted to a starting concentration of 1:25, serially diluted 1:2 in blocking solution, and incubated for 2 h at room temperature. The plates were washed three times with PBS-T, and 100 µl of competing anti-group 2 stalk biotinylated mouse MAb 9H10 (31) diluted to a concentration of 0.20 µg/ml in blocking solution was added to all wells. After 1 h, the plates were washed with PBS-T, and 50 µl of streptavidin labeled with HRP (Thermo Fisher catalog no. 21130) diluted 1:3,000 in blocking solution was added to all wells and incubated for 1 h at room temperature. The plates were washed four times with PBS-T and developed with OPD for 10 min, and the reaction was stopped with 3 M HCl (Thermo Fisher). The plates were read at a wavelength of 490 nm with a microplate reader (BioTek), and the data were analyzed in GraphPad Prism 7 and Microsoft Excel. The cutoff value was defined as the average of the values of blank wells plus 3 times the standard deviation of the blank wells. Percent competition was calculated based on the average signal of the mouse MAb 9H10-only wells on each plate.

### Microneutralization assay.

Serum samples were treated with receptor-destroying enzyme (RDE; Denka Seiken) for 18 h at 37°C. To stop RDE treatment, sodium citrate (2.5%) was added and serum was incubated at 56°C for 1 h. The inactivated serum samples (dilution of 1:10) were serially diluted 2-fold in UltraMDCK medium (Lonza), supplemented with tosyl phenylalanyl chloromethyl ketone (TPCK)-treated trypsin (infection medium; Sigma) at a concentration of 1 µg/ml, in 96-well cell culture plates (Sigma). The A/Shanghai/1/2013 H7N9 virus was diluted to a concentration of 100 50% cell culture infectious doses (TCID_50_) in infection medium. Sixty microliters of serially diluted serum was incubated with 60 µl of virus dilution (1,250 PFU/60 µl) for 1 h at room temperature on a shaker. MDCK cells were washed once with 220 µl of PBS, and 100 µl of the virus-serum mixture was added to MDCK cells. The cells were incubated for 48 h at 33°C. The readout was performed by the means of a hemagglutination assay. In brief, chicken red blood cells (RBCs; Lampire) were washed once with PBS and diluted to a concentration of 0.5% RBCs in PBS, and 50 µl of RBCs was added to 50 µl of cell supernatant in V-bottom plates (Corning). The plates were kept at 4°C for 30 to 45 min and scanned, and the results were analyzed in Microsoft Excel and GraphPad Prism 7.

### ADCC reporter assay.

MDCK cells (100 µl) at a concentration of 2 × 10^5^ cells/ml were seeded in white polystyrene 96-well plates (Costar Corning). The next day, the cells were washed once with PBS, and 100 µl of H7N9 A/Anhui/1/2013 virus diluted to a concentration of 2.8 × 10^5^ PFU/100 µl (multiplicity of infection [MOI] of about 1) in UltraMDCK medium (Lonza) was added to each well and incubated for 24 h at 37°C. In 96-well cell culture plates, sera (baseline and day 42 sera of 10 randomly selected individuals) were serially diluted 2-fold (1:10 starting concentration) in Roswell Park Memorial Institute (RPMI) 1640 medium (Life Technologies), and ADCC bioeffector FcγRIIIa cells (Promega) were thawed. The MDCK cells were washed once with 220 µl PBS, and 25 µl of RPMI 1640 medium, 25 µl of bioeffector FcγRIIIa cells (6.25 × 10^4^ cells/25 µl), and 25 µl of serially diluted sera were added. After incubation for 6 h at 37°C, 75 µl of Bio-Glo luciferase (Promega) was added to each well. The cells were incubated for 10 min in the dark before measuring the luciferase-induced luminescence with a microplate reader (BioTek). The results were analyzed in GraphPad Prism 7, and the AUC values were determined. The cutoff was defined as the average of the values of the blank wells plus 5 times the standard deviation of the blank wells.

### Passive transfer challenge experiments in mice.

Pre- (baseline) and postvaccination (day 42) serum samples of the different treatment groups were pooled separately, and 150 µl of the serum per pool was administered intraperitoneally to 6- to 8-week-old female BALB/c mice (10 mice per group). After 2 h, the mice were anesthetized with a ketamine-xylazine-water mixture (0.15 mg ketamine/kg of body weight and 0.03 mg/kg xylazine; 100 µl intraperitoneally) and challenged with 2 × 10^4^ PFU of H7N9 A/Shanghai/1/2013 virus (PR8 reassortant; corresponds to 5 50% murine lethal doses) in 50 µl PBS intranasally. All mice were bled to verify successful serum transfer by ELISA as previously described (65). Weight was monitored daily for 14 days, and a weight loss of 25% of initial weight was used as the humane endpoint. All procedures were performed in accordance with the Icahn School of Medicine at Mount Sinai Institutional Animal Care and Use Committee (IACUC) guidelines.

### Ethics statement.

The original clinical study was approved by the Western Institutional Review Board (Seattle, WA), and the protocol was registered at ClinicalTrials.gov (NCT02464163), carried out in accordance with the standards of the International Conference on Harmonization-Good Clinical Practices (ICH-GCP), and followed the ethical principles established in the Declaration of Helsinki. All subjects provided written informed consent prior to enrollment.

### Statistical analysis.

Statistical analysis was performed in GraphPad Prism 7. Data are shown as geometric means. Confidence intervals were calculated as 95% of the GM. Different time points and treatment groups were compared in a one-way analysis of variance (ANOVA) with a Sidak posttest for multiple comparisons. Detailed descriptions of groups compared are provided in the corresponding figure legends. The sequences for the phylogenetic tree were assembled in Clustal Omega and visualized in FigTree.

### Data availability.

The data that support the findings of this study are available from the corresponding author upon request.
