# Supplementary figures and images for: Vaccination with a Recombinant H7 Hemagglutinin-Based Influenza Virus Vaccine Induces Broadly Reactive Antibodies in Humans
Source: mSphere. 2017 Dec 13;2(6):e00502-17. doi: 10.1128/mSphere.00502-17 (PMC5729220; doi:10.1128/mSphere.00502-17)

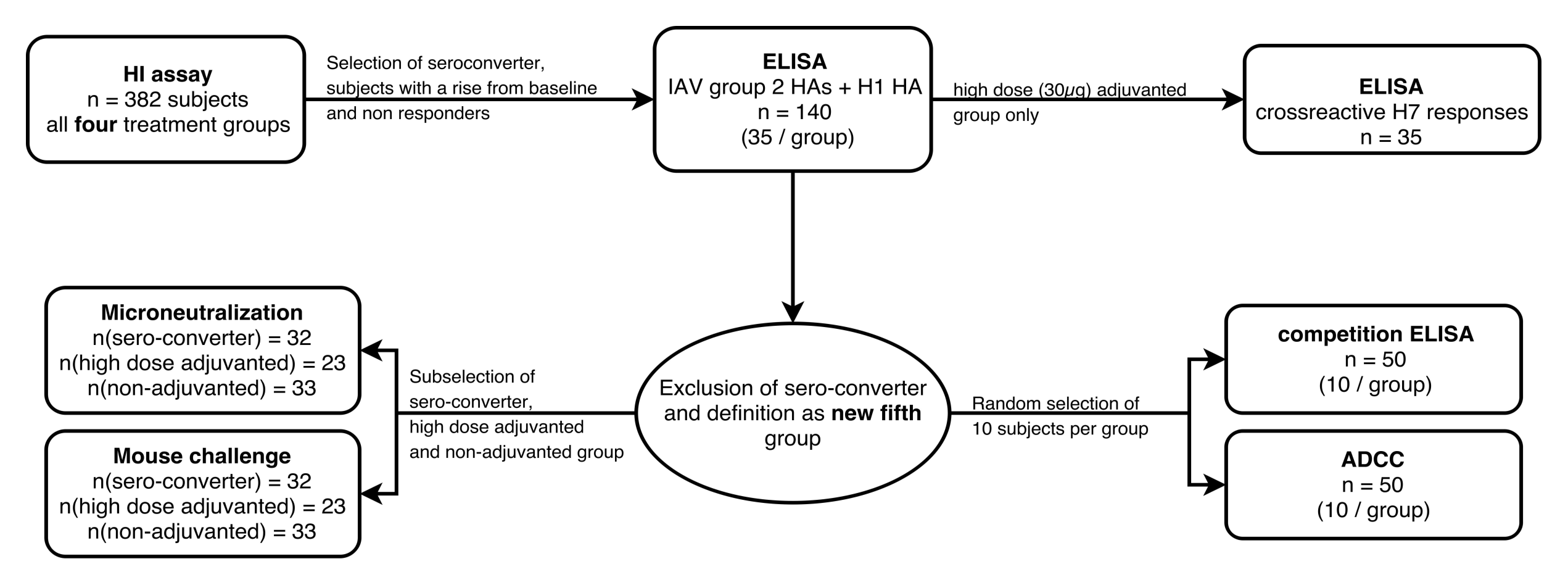

Supplement: FIG S1 [file sph006172428sf1.tif]

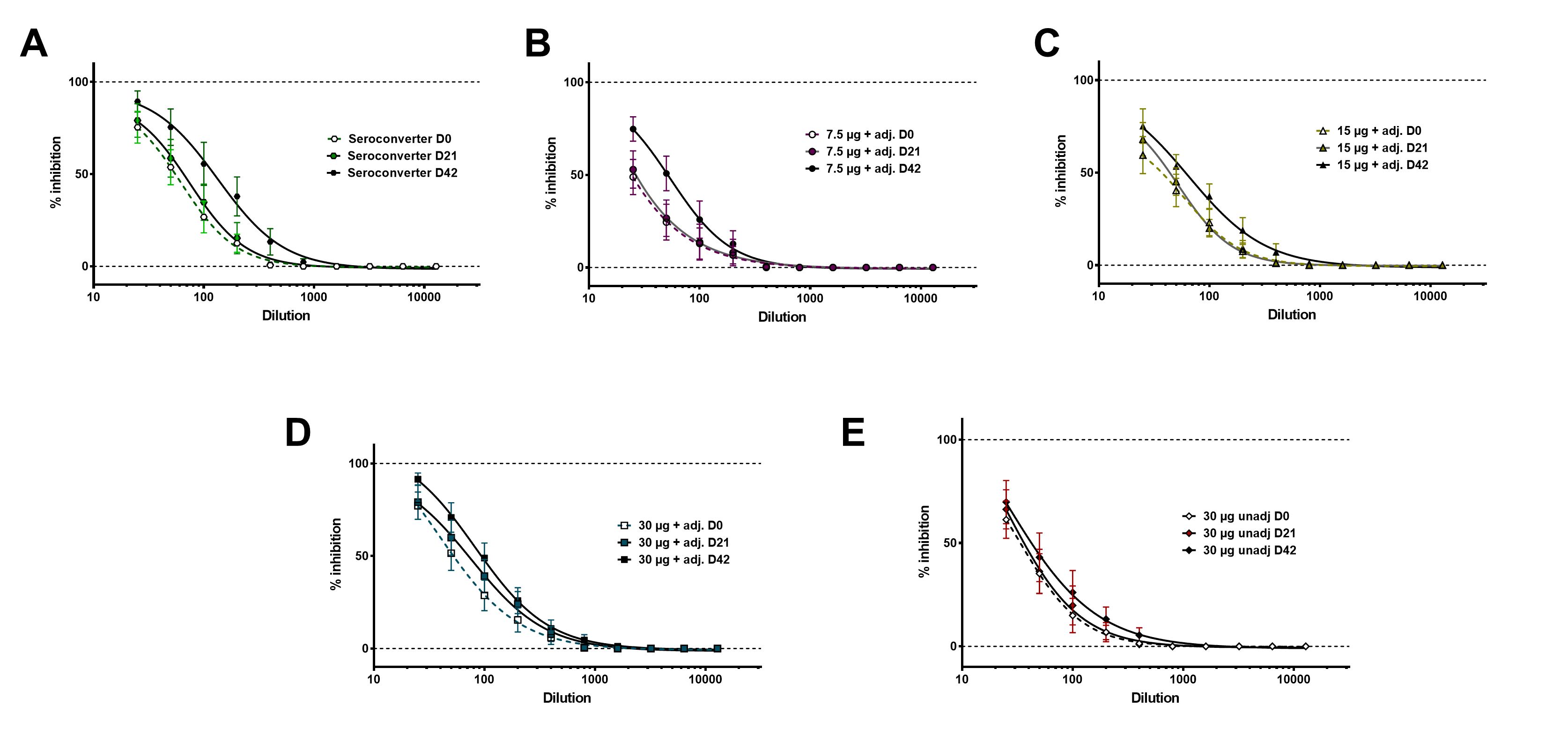

Supplement: FIG S2 [file sph006172428sf2.tif]
